# How Does Proline Treatment Promote Salt Stress Tolerance During Crop Plant Development?

**DOI:** 10.3389/fpls.2020.01127

**Published:** 2020-07-23

**Authors:** Ahmed El Moukhtari, Cécile Cabassa-Hourton, Mohamed Farissi, Arnould Savouré

**Affiliations:** ^1^ Sorbonne Université, UPEC, CNRS, IRD, INRA, Institut d’Ecologie et Sciences de l’Environnement de Paris, IEES, Paris, France; ^2^ Laboratory of Biotechnology & Sustainable Development of Natural Resources, Polydisciplinary Faculty, Sultan Moulay Slimane University, Beni Mellal, Morocco

**Keywords:** salinity, proline, plant development, photosynthesis, biological nitrogen fixation, nutrient uptake, water nutrition, antioxidants

## Abstract

Soil salinity is one of the major abiotic stresses restricting the use of land for agriculture because it limits the growth and development of most crop plants. Improving productivity under these physiologically stressful conditions is a major scientific challenge because salinity has different effects at different developmental stages in different crops. When supplied exogenously, proline has improved salt stress tolerance in various plant species. Under high-salt conditions, proline application enhances plant growth with increases in seed germination, biomass, photosynthesis, gas exchange, and grain yield. These positive effects are mainly driven by better nutrient acquisition, water uptake, and biological nitrogen fixation. Exogenous proline also alleviates salt stress by improving antioxidant activities and reducing Na^+^ and Cl^−^ uptake and translocation while enhancing K^+^ assimilation by plants. However, which of these mechanisms operate at any one time varies according to the proline concentration, how it is applied, the plant species, and the specific stress conditions as well as the developmental stage. To position salt stress tolerance studies in the context of a crop plant growing in the field, here we discuss the beneficial effects of exogenous proline on plants exposed to salt stress through well-known and more recently described examples in more than twenty crop species in order to appreciate both the diversity and commonality of the responses. Proposed mechanisms by which exogenous proline mitigates the detrimental effects of salt stress during crop plant growth are thus highlighted and critically assessed.

## Introduction

Salinity is a major abiotic stress that severely affects crop plant growth and development from seed germination to harvest. In recent years, increasing deleterious effects on agricultural productivity have been observed especially in arid and semiarid regions where rainfall is low and evapotranspiration is high (Jha et al., 2019). It is estimated that more than 7% of total land and almost 20% of arable land are affected by salinity with affected areas increasing at an annual rate of 1–2% (Zhu and Gong, 2014; Rizwan et al., 2015). It is indeed predicted that more than 50% of arable land will be rendered unproductive by 2050 due to the levels of salt stress induced in crops (Vinocur and Altman, 2005; Bianco and Defez, 2009). This trend coincides with the increasing challenge of ensuring global food security, so it is even more urgent to be able to exploit more arable land and increase crop productivity even in infertile soil by developing efficient and tolerant crops able to grow in salty conditions (Rengasamy, 2006; Safdar et al., 2019). Thus, new alternative approaches to allow crops to efficiently tolerate salt stress are needed. Indeed, the use of exogenous compounds, which are both ecofriendly and easily available, such as silicon (Zhu and Gong, 2014; Rizwan et al., 2015), trehalose (Nounjan et al., 2012), glycine betaine (Hossain and Fujita, 2010), and proline (Hoque et al., 2008; Deivanai et al., 2011; Wani et al., 2016), is a sustainable approach to overcoming the negative effects of salt stress on seed germination, plant growth, and productivity.

Proline is the most common endogenous osmolyte accumulated under various abiotic stresses including salinity (Szabados and Savouré, 2010; Slama et al., 2015). When applied as an exogenous compound to crops, proline can improve salt tolerance (Heuer, 2010). For example, in salt-stressed *Zea mays*, foliar application of proline increased plant growth with a positive effect on yield characteristics (Alam et al., 2016). The beneficial effects of exogenous proline application on salt stress tolerance has been the subject of several reviews. For example, Ashraf and Foolad (2007) focused on the effect of exogenous proline on seed germination, seedling growth and Na^+^/K^+^ ratio. More recently Meena et al. (2019) considered some beneficial effects of exogenous proline on plant tolerance to varying environments. Some of the latest progress in the subject addresses aspects related to ionic toxicity reduction, biological nitrogen fixation, and salt tolerance related-gene expression. Therefore, this review integrates this most recent research with current thinking on proline and plant salt tolerance in the context of some key developmental stages of crop growth.

## Impacts of Salinity on Developmental Physiology of Crop Plants

With the exception of halophytes, which represent 1–3% of the flowering plants, most plants, and especially crops, are salt-sensitive during their life cycle. Salt stress reduces plant growth and productivity (for review see van Zelm et al., 2020) and may be a direct effect due to the accumulation of Na^+^ and Cl^−^ or an indirect effect due to water deprivation (Ghars et al., 2008; Safdar et al., 2019).

**Figure 1:**
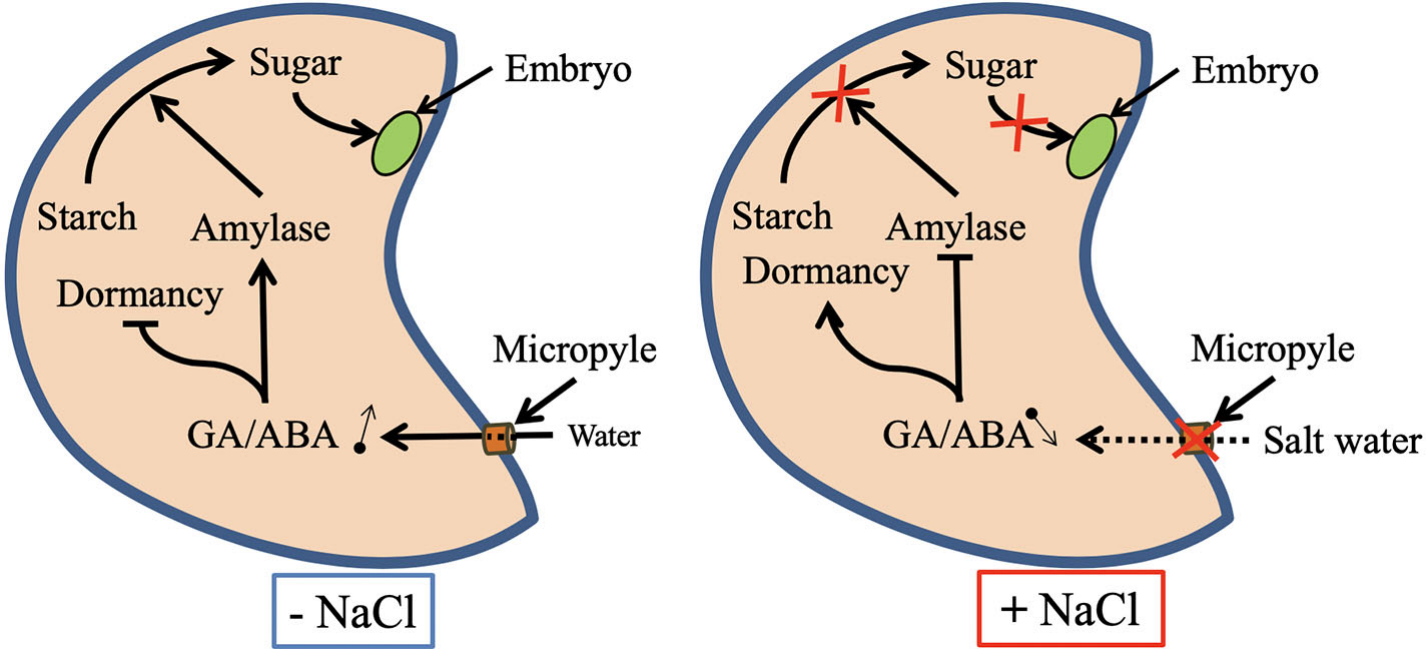
Proposed mechanisms for the regulation of seed dormancy release and seed reserve mobilization under salt-stressed and unstressed conditions. Abbreviations: ABA, abscisic acid; GA, gibberellic acid.

Seed germination may be drastically affected by salinity in both glycophytes and halophytes (Ghoulam and Fares, 2001; Liu et al., 2018). Salinity inhibits *Lens culinaris* seed germination by disturbing hydrolytic enzyme activities such as *α*-amylase, *β*-amylase and *α*-glucosidase (Sidari et al., 2008). Salt-inhibited *Medicago sativa* seed germination is correlated with the inhibition of the seed reserve mobilization (Farissi et al., 2011). Furthermore, salinity is known to inhibit seed germination by disturbing the homeostasis of plant growth regulators such as abscisic acid and gibberellic acid, the two of the main phytohormones participating in the regulation of germination (Holdsworth et al., 2008; Skubacz et al., 2016). Salinity causes secondary stress, known as oxidative stress, when reactive oxygen species (ROS) accumulate in cells. At high levels, ROS disturb normal metabolism by peroxidating proteins, lipids, and nucleic acids (Hayat et al., 2012; Farissi et al., 2014). Salinity-induced oxidative stress and membrane damage during germination and seedling growth have been described in several plant species and explain some of the deleterious effects of salt stress on seed germination (Wang et al., 2010; Zhang et al., 2015). The proposed effects of salinity on plant during seed germination are summarized in **Figure 1**.

**Figure 2:**
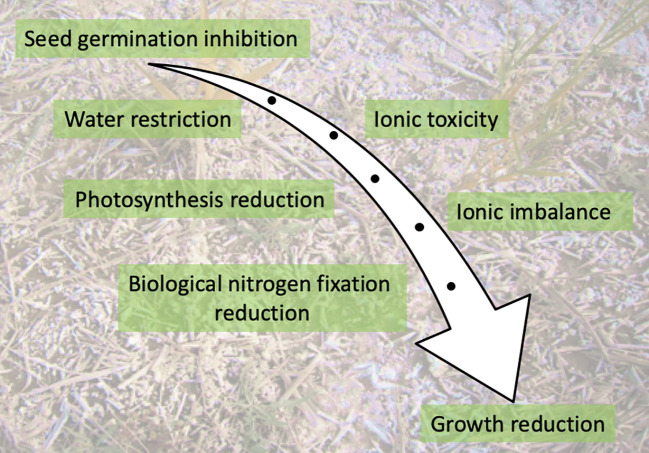
Key impacts of salinity on a developing plant.

As the radicle and later the roots emerge, the presence of salt triggers osmotic stress which makes water uptake more difficult. In addition, high salt concentrations in soil disrupt mineral nutrition leading to ion imbalance in the cells. Accumulation of excess sodium in plant cells has a toxic effect as it leads to precipitation or partial denaturation of proteins, phytohormone imbalances, generation of ROS, and changes in membrane permeability (**Figure 2**). Salinity was also reported to affect N metabolism at different steps including N uptake, NO_3_^−^ reduction and NH_4_^+^ assimilation by disturbing the activities of the main enzymes involved in nitrogen metabolism such as nitrate reductase, nitrite reductase, glutamate synthetase, and glutamate synthase (Ashraf et al., 2018).

As the seedling establishes itself to become autotrophic, photosynthesis, essential for growth, is very vulnerable to salt stress (**Figure 2**) (Safdar et al., 2019). Numerous studies have reported that photosynthesis is suppressed by salinity in several plant species. Smaller leaf area, fewer photosynthesis pigments, lower quantum efficiency of photosystem II (*Fv/Fm*) and less gas exchange were reported under salty conditions, which clearly contributed to the reduction observed in length and biomass of both shoots and roots (Ben Ahmed et al., 2011; Wani et al., 2016). Likewise, salinity was reported to induce the activity of some enzymes that degrade chlorophyll (Jamil et al., 2007). As a consequence of chlorophyllase induction, the total amount of chlorophyll decreases and chloroplast structure is disturbed, which directly influence photosynthesis rate and hence plant growth. Under osmotic stress, plants close their stomata to prevent water loss by transpiration (Yang et al., 2006). However, this mechanism also limits the assimilation of CO_2_, which then slows the photosynthesis rate and limits plant growth and productivity. High levels of salt may also affect cell division (Munns and Tester, 2008).

**Figure 3:**
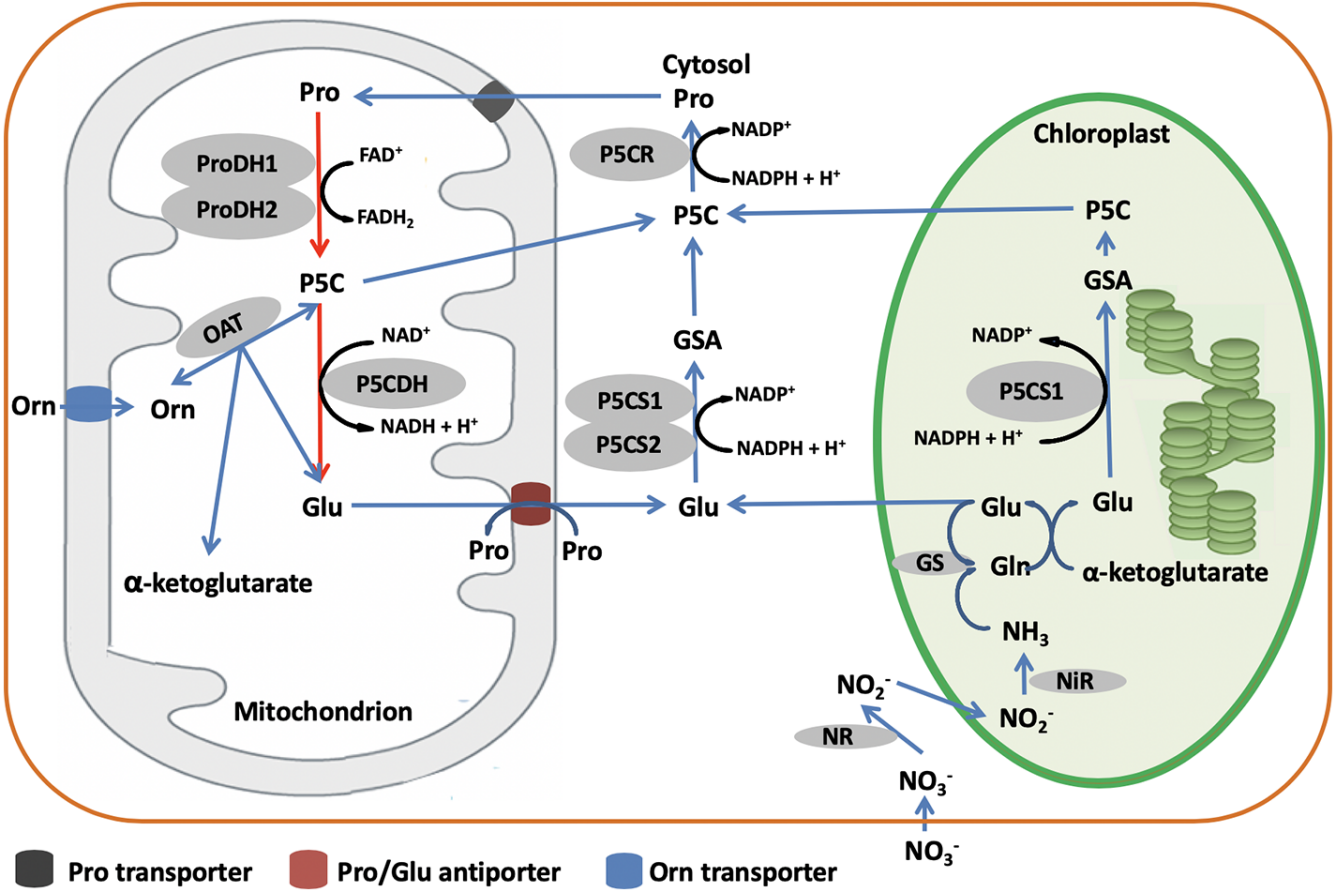
Proposed proline metabolism pathways in higher plants. Biosynthesis (blue lines) and catabolism (red lines) are shown. Abbreviations: Pro, proline; Glu, glutamate; Orn, ornithine; NR, nitrate reductase; NiR, nitrite reductase.

Legume–rhizobium symbiosis is a specific relationship established between legumes and nitrogen-fixing bacteria such as rhizobia. During this mutualistic symbiosis, inside a newly formed organ called a nodule, rhizobia are able to provide enough nitrogen to the host legume through the specific activity of nitrogenase and in return they receive a variety of carbon-based compounds from photosynthates and some micronutrients *e.g.*, Fe, S, Mo (Checcucci et al., 2017). Legume–rhizobium symbiosis represents one of the main ecological processes in the agroecosystem due to its benefits on soil fertility (Farissi et al., 2014). However, this symbiotic process is drastically limited by salt stress, affecting both the micro- and macro-symbiont. Indeed, depending on their sensitivity, salinity affects the survival and distribution of rhizobia in the soil (Zahran, 1999). Salinity was also reported to inhibit legume–rhizobium symbiosis establishment by reducing the number of root hairs containing infection threads (Zahran and Sprent, 1986). In addition, if symbiosis is already established, salinity decreases symbiotic performance by reducing leghemoglobin synthesis and nitrogenase activity (López et al., 2008). Studies also showed that salt stress limits the supply of carbon sources to the bacteroids by reducing the activity of phosphoenolpyruvate carboxylase and malate dehydrogenase, and so the number of bacteroids inside the nodule (López et al., 2008). The main effects of salt stress on legume–rhizobium symbiosis are summarized in **Figure 3**.

## Proline Metabolism in Plants

**Figure 4:**
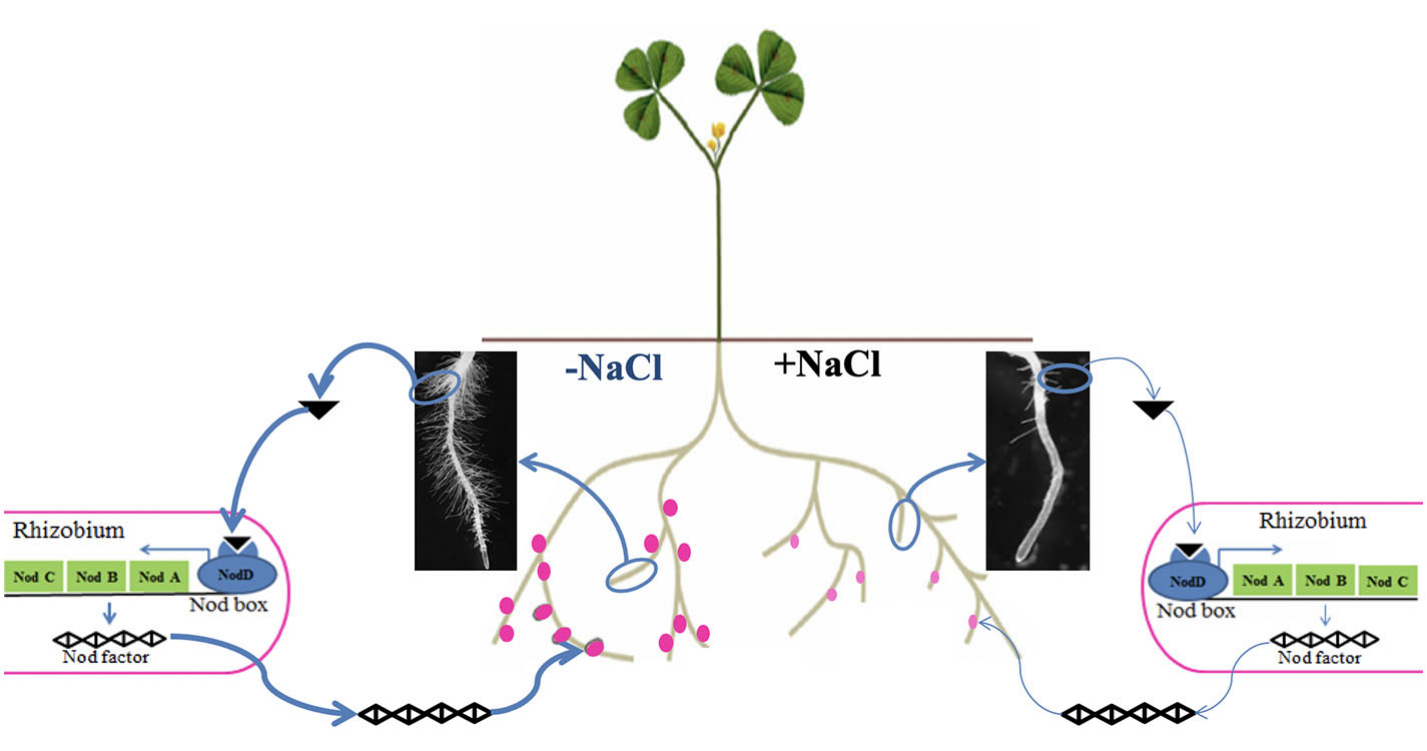
Conceptual illustration of the effect of salt stress on the establishment and functioning of legume–rhizobium symbiosis. Under nitrogen deficiency, without salt stress the legume emits many (bold arrow) chemical cues in the form of isoflavonoids (black triangle) to specifically attract rhizobia, which in turn secrete Nod factors allowing recognition between the two partners and formation of functional nodules (dark pink dots). However, salt stress negatively affects the legume–rhizobium dialogue by reducing isoflavonoid secretion (thin arrow) leading to less nodulation (number and biomass) and nodule function (pale pink dots).

In higher plants, biosynthesis of proline occurs *via* two pathways depending on the relative availability of the alternative substrates, glutamate (Glu) and ornithine (Orn) (**Figure 4**). The Glu pathway starts with pyrroline-5-carboxylate synthetase (P5CS) that uses ATP and NAD(P)H+H^+^ to reduce Glu to glutamate-semialdehyde (GSA), which spontaneously converts to pyrroline-5-carboxylate (P5C) (Szabados and Savouré, 2010). Then, P5C is reduced to proline by the action of P5C reductase (P5CR) using NADPH and H^+^ (Szabados and Savouré, 2010). In most plant species, P5CS is encoded by two genes, *P5CS1* and *P5CS2*, while P5CR is encoded by only one gene (Szabados and Savouré, 2010). However, in some species like *Medicago truncatula*, P5CS is possibly encoded by three genes (Kim and Nam, 2013; Nguyen et al., 2013). The Orn pathway has mostly been considered as an alternative pathway for proline biosynthesis. Ornithine-*δ*-aminotransferase (OAT) transaminates Orn to produce GSA and P5C, which is then reduced to proline by the action of P5CR (Mansour and Ali, 2017). According to You et al. (2012), transgenic lines of rice constitutively overexpressing *OAT* produce higher levels of proline than wild type, pointing to a more pivotal role of the Orn pathway in proline biosynthesis.

Although the genes and enzymes involved in proline biosynthesis have been well studied, the preferential use of Glu or Orn as substrate is still unclear. Some authors have reported that the preferred pathway is dependent on the developmental stage, the Orn pathway having a particularly crucial role in seedling development (Schmid et al., 2005; Hayat et al., 2012). Others however, have documented that the pathway preference is species-dependent. Indeed, AbdElgawad et al. (2015) noted that the Glu pathway involving P5CS and P5CR is predominant in grass, while the Orn pathway with OAT and P5CR is predominant in legumes. This difference may be related to the N nutritional status. In fact, Delauney et al. (1993) found that OAT is nitrogen-dependent. Another aspect to consider is the environmental control over which proline biosynthetic pathway is used. Zhen and Ma (2009) showed that P5CS activity (Glu pathway) increased upon salt stress treatment, while OAT activity (Orn pathway) appeared not to be affected, suggesting that the Glu pathway rather than the Orn pathway plays a more significant role in proline accumulation during osmotic regulation. In *Vigna aconitifolia*, Delauney et al. (1993) showed that salt stress induced the accumulation of *P5CS* mRNA while *OAT* mRNA levels were suppressed. Lei et al. (2016) and Mansour and Ali (2017) confirmed that the accumulation of proline under salt stress is related to the up-regulation of *P5CS* (Glu pathway) genes and down-regulation of *Proline dehydrogenase* (*PDH*) genes. In comparison, Funck et al. (2008) demonstrated that OAT is localized in mitochondria and it is not essential for proline biosynthesis.

For catabolism, proline is converted back to Glu in the mitochondria by the sequential action of PDH and P5C dehydrogenase (P5CDH). Although the Nomenclature Committee of The International Union of Biochemistry and Molecular Biology (IUBMB) recommended the name glutamate *γ*-semialdehyde dehydrogenase (GSALDH), an enzyme name derived from its substrate, for the second enzyme of proline catabolism, the name P5CDH is kept in the review for clarity for the research community. PDH oxidizes proline to P5C which is converted to Glu by P5CDH using NAD^+^ as electron acceptor (Servet et al., 2012; Schertl et al., 2014). In some plant species including *Arabidopsis thaliana*, *Nicotiana tabacum*, and *M. sativa*, PDH is encoded by two genes, whereas P5CDH is encoded by a single gene (Miller et al., 2005; Ribarits et al., 2007; Servet et al., 2012).

## Influences of Proline Metabolism on Physiological and Biochemical Processes

**Figure 5:**
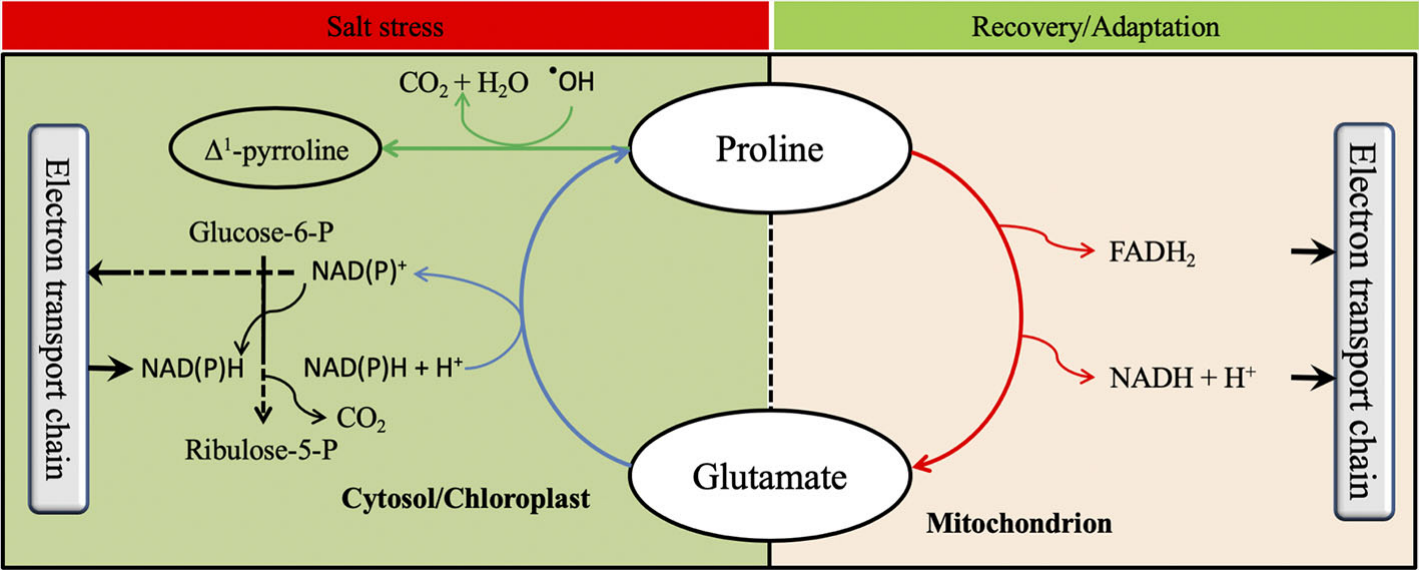
Possible influence of proline metabolism on physiological and biochemical processes. The oxidative pentose phosphate pathway is shown by black lines, the proline anabolic pathway with blue lines, the proline catabolic pathway with red lines, and proline scavenging reactive oxygen species with green lines.

Proline has been widely reported to be a multifunctional amino acid that acts at different plant growth stages (Szabados and Savouré, 2010). Indeed, proline metabolism plays a key role in the oxidative pentose phosphate pathway (OPPP) by generating NAD(P)^+^ in the cytosol (Signorelli, 2016). Since the OPPP is involved in triggering seed germination, it is believed that proline metabolism has a beneficial effect on seed germination (Kavi Kishor and Sreenivasulu, 2014). When stomata are closed under osmotic stress to avoid water losses by transpiration, CO_2_ assimilation is limited (Yang et al., 2006). This phenomenon reduces carbon fixation and NAD(P)H consumption by the Calvin cycle and leads to accumulation of ROS by electrolyte leakage. However, proline biosynthesis requires the oxidation of two NAD(P)H^+^ molecules to NADP^+^ (**Figure 4**), which helps to reduce NAD(P)H and recycle NAD(P)^+^. Furthermore, the oxidation of NAD(P)H to NADP^+^ during proline biosynthesis increases NADP^+^ which will be reduced in the pentose phosphate pathway to NAD(P)H^+^ generating one molecule of CO_2_ (**Figure 5**). Thus, the CO_2_ generated allows carbon reduction to continue under stressful conditions, while the NAD(P)H will be used in proline biosynthesis to prevent ROS production (Verslues and Sharma, 2010; Signorelli et al., 2015). Proline was also reported to contribute to photosynthesis improvement by protecting RuBisCo activity and mitochondrial electron transport chain complex II (Solomon et al., 1994; Hamilton and Heckathorn, 2001). Furthermore, proline anabolism allows plants to adjust their osmotic homeostasis which helps to restore plant water content particularly under osmotic stress (Misra and Gupta, 2005). Proline metabolism has also been documented to play an important role during biological nitrogen fixation (BNF) particularly under stressed conditions. Indeed, a high positive correlation between the expression of *StP5CS* and two nodulation-related genes and one leghemoglobin gene was reported by Ren et al. (2018). Likewise, proline catabolism was reported to provide energy to the bacteroids during the BNF (Kohl et al., 1988) suggesting that both proline anabolism and catabolism improved BNF efficiency. In addition, proline has been reported in several studies to play a role in non-enzymatic antioxidant activities (Matysik et al., 2002; Signorelli et al., 2015). These proposed roles of proline metabolism in key physiological and biochemical are illustrated in **Figure 5**.

## Effect of Exogenous Proline During Salt Stress

### Exogenous Proline Application and Proline Metabolism Under Salt Stress

Many studies show that salt stress triggers the induction of genes involved in proline biosynthesis, which leads to proline accumulation (Armengaud et al., 2004; Kim and Nam, 2013; Nguyen et al., 2013). According to Székely et al. (2008), knocking out the function of P5CS in *A. thaliana* indicates a key role for this enzyme in plant salt tolerance because the *p5cs1* plants are hypersensitive to salt. Exogenous application of proline can effectively improve tolerance of plants to salt stress through the regulation of endogenous proline metabolism, partly achieved through differential expression of specific proline-related genes. For example, de Freitas et al. (2018) demonstrated that foliar application of proline to *Z. mays* resulted in a decrease in P5CS activity and an increase in PDH under salt stress. Similar results in salt stressed *Sorghum bicolor* were reported more recently (de Freitas et al., 2019). Adding exogenous proline led to a decrease in P5CS activity in both stressed and unstressed *Eurya emarginata*, but to an increase in PDH activity only in unstressed plants (Zheng et al., 2015). Under salt stress, *Triticum aestivum* seed priming with exogenous proline significantly decreased the content of proline and P5C with a reduction in the activity of P5CS, while PDH activity was significantly increased (Rady et al., 2019). The effect of exogenous proline on *PDH* expression was also reported by Kiyosue et al. (1996) and Nakashima et al. (1998) in *A. thaliana*. Deuschle et al. (2001) reported that, in addition to PDH, exogenous proline increased *P5CDH* transcript levels, and suggested that this response may protect plants against proline toxicity. However, other authors like Nounjan et al. (2012) have shown that applying exogenous proline significantly increased expression of *P5CS* and *P5CR* in salt-stressed *Oryza sativa*.

### Effect of Proline Treatment on Seed Germination Under Salt Stress

Seed germination is one of the most critical stages in the plant life cycle (Hubbard et al., 2012) because it is very sensitive to abiotic stress. In particular, salt stress causes osmotic stress that limits seed water absorption and ion toxicity due to the high accumulation of Na^+^ and Cl^−^ (Murillo-Amador et al., 2002; Farissi et al., 2011). In recent years, there have been numerous papers about the effect of exogenous compounds like hormones, mineral elements, and amino acids in alleviating salinity stress during seed germination (Atia et al., 2009; Dallali et al., 2012; Rizwan et al., 2015; Coskun et al., 2016). However, the effect of exogenous proline on seed germination under salt stress is poorly understood as only a few studies have been published. Deivanai et al. (2011) demonstrated that exogenous proline had a positive concentration-dependent effect on seed germination under salt stress. Application of 1 mM proline alleviates the negative effect of 400 mM NaCl, but 100 mM proline did not have a significant effect. Similarly, 50 mM proline treatment improved seed germination of two cultivars of *S. bicolor* under salt conditions (Nawaz et al., 2010). Therefore exogenous proline application at suitable concentrations may alleviate the negative effect of salt stress by regulating cellular osmotic balance, but detailed studies behind these data are still needed to better understand the molecular mechanisms involved.

### Effects of Proline Treatment on Plant Growth and Biomass Under Salt Stress

It is well documented that certain concentrations of exogenous proline regulate different aspects of plant growth and development under salt stress including rises in biomass and productivity (Huang et al., 2009; Nawaz et al., 2010; Nounjan et al., 2012; Wu et al., 2017). Addition of exogenous proline improved the growth of calli from two *Medicago sativa* cultivars upon salt stress, but dry weight and proline contents between the two were different with a better salt tolerance correlated with higher proline accumulation (Ehsanpour and Fatahian, 2003). Khan et al. (2014) tested the effects of 30 and 60 mM proline applied as a foliar spray to *Helianthus annuus*, concentrations that induced tolerance to 60 and 120 mM NaCl. They found that exogenous proline mitigates the salt stress effects on plant growth as proven by longer shoots and roots, and greater fresh and dry weights of shoots and roots, and this positive effect was more pronounced at the lower proline concentration (30 mM). Similarly, Wani et al. (2016) reported that a foliar spray of 20 mM proline alleviates the negative effects of salt stress on *Brassica juncea* by increasing lengths and fresh and dry masses of both shoots and roots, and the area of leaves. In addition, exogenous proline supply significantly increased plant height and number of roots in salt stressed *O. sativa* (Teh et al., 2016). Likewise, application of proline increased dry mass of leaves and roots and their soluble protein contents in salt stressed *Z. mays* (de Freitas et al., 2018). In some cases, exogenous proline stimulates yield under salt stress. Exogenous proline increased fresh and dry biomasses, grain yield and 1000-grain weight of salt-stressed *T. aestivum* (Rady et al., 2019). In salt-stressed *Z. mays*, foliar-applied proline increased the number of seeds per plant, total grain weight and the 100-grain weight (Alam et al., 2016). In general, exogenous application of proline increased plant growth and productivity under salt-induced stress but the underlying mechanisms, probably linked to some hormonal regulation, still remain elusive.

### Exogenous Proline Alters Stress-Responsive Gene Expression Under Salt Stress

Evidence for the mechanisms by which exogenous proline improves plant salt tolerance is still scarce. In order to gain some insight into such mechanisms at the gene level, Nounjan et al. (2012) studied the effect of exogenous proline on the expression of proline metabolism-related genes *P5CS* and *P5CR* as well as genes encoding antioxidant enzymes, superoxide dismutases (*Cu/ZnSOD, MnSOD*), ascorbate peroxidase (*CytAPX*), and catalase (*CatC*), in salt-stressed *O. sativa* seedlings. Results showed that after six days of salt treatment, exogenous proline upregulated *P5CS* and *P5CR* transcript levels. Likewise, the genes encoding antioxidant-related enzymes were upregulated by exogenous proline added to the salt-stressed rice plants. In a different study on salt-stressed *N. tabacum*, exogenous proline was found to increase transcript levels of genes encoding SOD, cationic peroxidase (POX) and CAT (Hoque et al., 2008). To understand more about the mechanistic role of gene regulation in exerting the effect of exogenous proline as plant salt tolerance, additional genetic experiments are required to particularly investigate the expression of genes related to the transport and translocation of Na^+^ and Cl^−^. More needs to be known about the relationship between the addition of proline and the expression of aquaporin-related genes under salt stress.

### Exogenous Proline Influences Plant–Water Relations Under Salt Stress

Much research has documented how exogenous proline substantially alleviates salt stress by increasing leaf water potential, water content and restoring water use efficiency (**Table 1**). In *Brassica juncea*, Wani et al. (2016) noted that the leaf water potential was reduced under salt stress, but 20 mM proline applied as a foliar spray completely reversed the loss in water potential. Similarly, Huang et al. (2009) demonstrated that, under saline conditions, exogenous proline could alleviate the growth inhibition of salt-sensitive *Cucumis sativus*, and this was accompanied with leaves having higher water content. Studying salt-stressed *O. europaea* plants, Ben Ahmed et al. (2011) found that the relative water content is 1.05 and 1.09-fold higher under 25 and 50 mM of exogenous proline, respectively, than in the absence of proline. In the same way, 20 mM exogenous proline significantly alleviated the negative effects of 200 mM NaCl and raised the leaf water content in *Eurya emarginata* (Zheng et al., 2015). The role of exogenous proline in maintaining higher plant water content under salinity was also reported in *Onobrychis viciifolia* (Wu et al., 2017) and *S. bicolor* (de Freitas et al., 2019).

**Table 1:** Effects of exogenous proline on seed germination, plant growth, photosynthesis, nutrient acquisition, water uptake, ionic toxicity, proline metabolism, gene expression, antioxidant activities, and biological nitrogen fixation in different plant species under salt stress.

Plant name	Salt concentration	Exogenous proline concentration	Application method	Variable	Effect of exogenous proline		
					Without stress	With stress	References
*Cucumis melo*	150 mM	10 mM	Foliar spray	Growth		+	Kaya et al. (2007)
				Chlorophyll content		+	
				Electrolyte leakage		+	
				Proline content	Not shown	+	
				Relative water content		+	
				Stomatal density		+	
				Nutrient acquisition and Na/K ratio		+	
	100 mM	10 mM	Foliar spray	Growth	–	+	Huang et al. (2009)
				Proline content	+	+	
*Cucumis*				Relative water content	–	+	
*sativus*				MDA	+	+	
				Antioxidant enzyme activities	+	+	
				Na^+^, Cl^-^ and K^+^ content	–	–	
	200 mM	10 mM	Nutrient solution	Growth	–	–	Zheng et al. (2015)
				MDA	–	+	
*Eurya*				Na^+^/K^+^ ratio	–	+	
*emarginata*				Antioxidant enzyme activities	–	+	
				P5CS activity	–	+	
				PDH activity	+	–	
	15 mM	25 mM	Not shown	Number of nodules	–	+	Sabagh et al. (2017)
*Glycine*				Biological nitrogen fixation	–	+	
				Nitrogenase activity	+	+	
*Helianthus annus*	60 mM 120 mM	30 mM 60 mM	Foliar spray	Growth Chlorophyll content Na+ and K+ content Nitrate reductase activity Protein content Total amino acids Total sugars	+ + + - + + --	+ + + + + + -	Khan et al. (2014)
*Mung bean*	300 mM	15 mM	Nutrient solution	Glutathione Antioxidant enzyme activities MDA and H2O2	Not shown		Hossain and Fujita (2010)
*Nicotiana tabacum*	200 mM	20 mM	Medium solution	Non enzymatic antioxidant activities Antioxidant enzyme activities Carbonyl content	Not shown	+ + +	Hoque et al. (2008)
*Olea europaea*	100 mM 200 mM	25 mM 50 mM	Nutrient solution	Relative water content and leaf water potential Gas exchange Photosynthetic pigment Compatible solute Mineral ion contents Na^+^/K^+^ and Na^+^/Ca2^+^ ratio	Not shown	+ + + + - +	Ben Ahmed et al. (2011)
*Onobrychi sviciaefolia*	25 mM 100 mM	2.5 mM	Nutrient solution	Growth Water content MDA Na^+^/K^+^ ratio Proline content	- - - - +	+ + - + +	Wu et al. (2017)
*Oryza sativa*	100 mM 200 mM 300 mM 400 mM	1 mM 5 mM 10 mM	Seed pretreatment	Seed germination Growth Chlorophyll content Proline content Protein content	- - + + +	+ + + - +	Deivanai et al. (2011)
	100 mM	10 mM	Nutrient solution	Growth Na^+^/K^+^ ratio Proline content H2O2 content Antioxidant enzyme activities *P5CS* gene expression *P5CR* gene expression Antioxidant enzyme gene expression	- + + + + + + +	- + + + + + + +	Nounjan et al. (2012)
	150 mM	5 mM 10 mM	Growth medium	Growth Nitrogen-metabolism enzyme activities Nitrogen content	+ - -	+ + +	Teh et al. (2016)
*Pisum sativum*	100 mM	60 mM	Foliar spray	Growth Gas exchange Chlorophyll content Relative water content Compatible solute H2O2, MDA and electrolyte leakage	+ + + + + -	+ + + + + +	Shahid et al. (2014)
*Sorghum bicolor*	75 mM	30 mM	Foliar spray	Growth Membrane damage Relative water content Gas exchange Nutrient uptake K^+^/Na^+^ ratio Amino acids Proline content P5CS activity OAT activity ProDH activity *P5CS* gene expression *OAT* gene expression *ProDH* gene expression	- - - - - - - + - - + - + +	+ + + + + + + + + - + + + +	de Freitas et al. (2019)
*Triticum durum*	120 mM	12 mM	Seed pretreatment	Growth Photosynthetic activities K^+^/Na^+^ ratio Proline content Proline metabolism enzyme activities MDA and H2O2 content Antioxidant enzyme activities Non-enzymatic antioxidant activities	- - - - - - - -	+ + + + + + + +	Rady et al. (2019)
*Zea mays*	25 mM 50 mM	25 mM 50 mM 100 mM	Foliar spray	Growth Grain yield Chlorophyll Nutrient uptake K^+^/Na^+^ ratio	Not shown	+ + + + +	Alam et al. (2016)
	80 mM	30 mM	Foliar spray	Growth Ion content K^+^/Na^+^ ratio Proline content P5CS activity ProDH activity Antioxidant enzyme activities Non enzymatic antioxidant activities MDA and H2O2 content	- - - + - + - - -	+ - - + + + + - +	de Freitas et al. (2018)

+ and - indicate positive and negative effects, respectively.

Many authors have suggested that the increase in water content and water potential of leaves in response to exogenous proline under salt stress could be because the proline triggers the accumulation of some organic and inorganic compounds such as proline, glycine betaine, soluble sugars and K^+^ that help plants adjust their cellular osmotic potential and hence maintain higher water content (Ben Ahmed et al., 2011; Nounjan et al., 2012; Khan et al., 2014; Zheng et al., 2015). Another possibility is that maintaining a favorable water content under osmotic stress may be attributed to the regulation of the expression of root aquaporin genes in response to exogenous proline. These possible mechanisms for mediating osmotic stress tolerance and improving plant water content need to be studied in more detail at the molecular level.

### Exogenous Proline Balances Mineral Nutrient Uptake and Assimilation Under Salt Stress

Salinity not only increases Na^+^ and Cl^−^ in plants but also induces decreases in Ca^2+^, K^+^, Mg^2+^, NO_3_^−^, S, and other essential nutrients leading to overall nutrient deficiency (Manchanda and Garg, 2008; Farissi et al., 2014). The positive effects of exogenous proline on plant tolerance to salt stress have been linked to increased assimilation of nutrients in many studies. Abdelhamid et al. (2013) reported that exogenous proline application increased P, K, NO_3_^−^ and NO_2_^−^ contents in *Phaseolus vulgaris* under different levels of salinity (three fields with electrical conductivities of 1.84, 6.03, or 8.97 dS m^−1^). Similarly, exogenous proline increased leaf N, Ca^2+^ and K^+^ contents in *Cucumis melo* exposed to stress from 150 mM salt (Kaya et al., 2007). Also under salty conditions, exogenous proline increased Ca^2+^ and K^+^ in *S. bicolor* (de Freitas et al., 2019) and *O. europaea* (Ben Ahmed et al., 2011). Alam et al. (2016) suggested that exogenous proline may increase the uptake of N, P, K^+^ and S in *Z. mays* under salinity. As well as nutrient uptake, the activities of some enzymes involved in nutrient assimilation are triggered by exogenous proline under salty conditions. Nitrate reductase is one of the most important enzymes involved in nitrogen assimilation and exogenous proline stimulates its activity in *H. annuus* (Khan et al., 2014) and *C. melo* (Yan et al., 2011) exposed to salt stress. Recently, Teh et al. (2016) reported that exogenous proline alleviated the negative effects of salt stress and enhanced nitrate reductase and Glu synthase activities in *O. sativa*. Some authors have suggested that proline may provide a good way to store and recycle nitrogen under stress conditions (Heuer, 2010; Szabados and Savouré, 2010; Verslues and Sharma, 2010; Ben Rejeb et al., 2014; Mansour and Ali, 2017). Consistent with this line of reasoning is evidence that PDH is stimulated in *P. vulgaris* under nitrogen deficiency suggesting that proline may be used as a nitrogen source for growth (Hayat et al., 2012). Similarly, exogenous proline was also used as a source of nitrogen by *Vigna radiata* L. seedlings under stress conditions (Posmyk and Janas, 2007).

The above studies provide preliminary evidence that exogenous proline alleviates the negative effects of salt by improving uptake of some nutrients as well as stimulating the activity of some enzymes involved in nutrient assimilation. However, research into the effect of exogenous proline on the translocation of micronutrients is limited.

### Proline Treatment Mediates Reduction in Ion Toxicity Due to Salt Stress

High salt concentrations increase Na^+^ and Cl^−^ contents in plants and decrease the abundance of other cations such as K^+^ and Ca^2+^, which leads to mineral nutrient imbalance (Zhu and Gong, 2014). Indeed, under salty conditions, sustaining ion homeostasis is one of the adaptive strategies that tolerant plants use to cope with salt stress. These strategies may help the plant to prevent potentially toxic effects of the build-up of ions like Na^+^ and Cl^−^ that cause various types of damage to lipids, proteins and nucleic acids (Zhu and Gong, 2014; Bargaz et al., 2015; Rizwan et al., 2015). Application of 5 mM proline in a foliar spray decreased Na^+^ content and increased K^+^/Na^+^ ratio in *P. vulgaris* (Abdelhamid et al., 2013). More recently, de Freitas et al. (2018) reported that external application of proline decreased both Na^+^ and Cl^−^ contents, but increased the K^+^ content and the K^+^/Na^+^ ratio in salt-stressed *Z. mays*. Similar results have been reported in *S. bicolor* (de Freitas et al., 2019). Khan et al. (2014) demonstrated that exogenous proline alleviated the negative effect of 120 mM salt, and enhanced K^+^ content, and reduced Na^+^ concentration in *H. annuus*. In salt-stressed *O. europaea*, exogenous proline improved salt tolerance through maintaining a low Na^+^ content, a high K^+^ content and lowered Na^+^/K^+^ and Na^+^/Ca^2+^ ratios in both young and old leaves (Ben Ahmed et al., 2011). Compared to salt-stressed plants, exogenous proline application increased the K^+^/Na^+^ ratio in *O. sativa* under 100 mM NaCl (Sobahan et al., 2012) and in *Z. mays* under 50 mM NaCl (Alam et al., 2016). Recently, Wu et al. (2017) reported that 2.5 mM exogenous proline decreased the Na^+^/K^+^ ratio in *Onobrychis viciifolia* Scop under 100 mM NaCl.

Removing Na^+^ from the cytosol and compartmentalizing it in the vacuole are important strategies to maintain a low Na^+^ concentration (Bargaz et al., 2015). Transgenic *Saccharum officinarum* overexpressing the *P5CS1* gene had a low Na^+^ content compared to wild type (Guerzoni et al., 2014). Ben Ahmed et al. (2011) had previously suggested that the lower accumulation of Na^+^ in proline-treated *O. europaea* under salt stress may be due to the effect of exogenous proline on the ability of root to exclude the salt ions Na^+^ and Cl^−^ from the xylem to the shoot. The activity of some transporters, like a plasma membrane Na^+^/H^+^ antiporter encoded by the *SALT overly sensitive* (*SOS*) gene, facilitates the export of Na^+^ from the cytosol to the leaves, protecting the plant from its toxicity (Zhu, 2003; Bargaz et al., 2015). Proline does not always act in this way to induce salt tolerance. Indeed, in *C. sativus*, exogenous proline has no significant effect on Na^+^ and K^+^ concentrations in leaves but improves leaf water content under 100 mM NaCl (Huang et al., 2009). This higher water content due to the exogenous application of proline may dilute the salt and therefore limit salt toxicity leading to better plant growth. This was confirmed by Ben Ahmed et al. (2011) who reported that the large reduction in Na^+^ accumulation in leaves and roots in response to exogenous proline application was due to its interference in osmotic adjustment and/or its dilution. Additional studies on the effect of exogenous proline on membrane transporters, such as Na^+^/H^+^ antiporters and K^+^/H^+^ symporters, are needed to investigate the mechanism by which exogenous proline reduces salt ion toxicity.

### Exogenous Proline Improves Photosynthesis Under Salt Stress

Abiotic stresses, including salt stress, cause stomata to close and chlorophyll synthesis to slow down (Hayat et al., 2012), while activating chlorophyllase activities (Jamil et al., 2007), damaging chloroplast structure and destabilizing pigment protein complexes (Singh and Dubey, 1995). These effects lead to a reduction in photosynthesis and, as a result, plant growth inhibition (Farissi et al., 2018). The beneficial effect of exogenous proline on plant growth under salt stress has often been associated with a change in photosynthesis parameters (**Table 1**) (Hayat et al., 2012; Mansour and Ali, 2017). Ben Ahmed et al. (2011) found that proline supplements to two-year-old *O. europaea* exposed to 100 or 200 mM NaCl resulted in higher levels of net photosynthesis, chlorophyll *a* and *b* and carotenoid contents as compared to salt-stressed plants without supplements. In a similar study, Wani et al. (2016) reported that exogenous proline increased various photosynthetic attributes including net photosynthesis, leaf area, stomatal conductance, intercellular CO_2_, transpiration rate, and quantum efficiency of photosystem II (Fv/Fm) in two salt-stressed *B. juncea* cultivars. Similar results were obtained in *Solanum melongena* (Shahbaz et al., 2013) and in *Pisum sativum* (Shahid et al., 2014). Nawaz et al. (2010) also reported a positive effect of exogenous proline on chlorophyll *a* and total chlorophyll contents in salt-stressed *S. bicolor*. However there was no equivalent significant difference in chlorophyll b content under 50 and 100 mM of NaCl. These findings strongly suggest that exogenous proline influences plant growth under salt stress by enhancing photosynthetic processes.

### Exogenous Proline Application Reduces Oxidative Stress in Salt-Stressed Plants

ROS are continuously generated in stressed plants due to the incomplete reduction of oxygen. Some of them can play a role as second messengers to trigger tolerance to abiotic stresses (Ben Rejeb et al., 2014). Proline has been considered to be a molecular chaperone due to its capacity to scavenge ROS, to stabilize protein and other macromolecular complexes, and to provide cellular redox potential (Szabados and Savouré, 2010; Ben Rejeb et al., 2014). Furthermore, under salt stress, exogenous proline increases enzymatic and non-enzymatic antioxidant activities, which improves plant tolerance. Indeed, Hossain and Fujita (2010) reported that exogenous application of 15 mM proline to the growth medium of mung bean exposed to 300 mM NaCl significantly decreased malondialdehyde (MDA) and H_2_O_2_ contents, and this decrease correlated significantly with an increase in glutathione content and glutathione peroxidase, glutathione-S-transferase and glutathione reductase activities. In the previously cited study by Wani et al. (2016), 20 mM proline sprayed on two *B. juncea* cultivars growing under three different concentrations of salt (2.8, 4.2, and 5.6 dS.m^−1^), reduced electrolyte leakage and increased the activities of some antioxidant enzymes like CAT, SOD and POX. At the same time, proline itself can contribute to ROS scavenging and hence to plant salt tolerance, including when it is supplied exogenously (Ben Rejeb et al., 2014). However, Nounjan et al. (2012) showed that exogenous application of 10 mM proline to salt stressed *O. sativa* seedlings decreased the activity of SOD, POX, and CAT and increased H_2_O_2_ content. In agreement with those results, Huang et al. (2009) reported that foliar spray of proline lowered the MDA content and the SOD activity in a salt-sensitive *C. sativus* cultivar under 100 mM NaCl. An increase in POX activity in response to exogenous proline was also measured in the salt-stressed cucumber. Mansour and Ali (2017) suggested that the decrease in antioxidant activities under salt stress in response to exogenous proline may be involved in the improvement of salt tolerance through ROS signaling. Species-specific differences may explain these contradictory results on proline effects.

### Symbiotic Nitrogen Fixation Is Enhanced by Proline Treatment Under Salt Stress Conditions

Soil inorganic nitrogen deficiency is one of the most limiting factors for plant growth. However, the biological reduction of atmospheric nitrogen to ammonium by rhizobia-legume symbiosis can provide enough nitrogen to maximize growth and yield (Zahran, 1999; Kraiser et al., 2011). Encouraging rhizobia–legume symbiosis is a sustainable approach to increasing crop production, while decreasing dependency on chemical nitrogen fertilizer in traditional agriculture, which causes widespread environmental pollution (Ferguson et al., 2019). Salt stress limits the distribution, survival, and infectivity of rhizobia by decreasing the number and the biomass of nodules, and diminishing leghemoglobin synthesis and nodule respiration leading to a decrease in nitrogenase activity and nitrogen fixation rate (Zahran, 1999; Faghire et al., 2011; Monica et al., 2013). Improving BNF under salt stress is considered to be a major goal for crop scientists. Several strategies have been adopted to improve BNF under high-salt conditions including the selection of the most tolerant rhizobium–legume combinations, use of arbuscular mycorrhizal fungi, improvement of agricultural practice, genetic breeding and plant genetic modification, seed priming and exogenous application of compounds like hormones and osmoprotectants (Faghire et al., 2011; Faghire et al., 2013; Sabagh et al., 2017; Farooq et al., 2017).

Although positive correlations between endogenous proline and BNF under salt stress have been reported in many studies (Tejera et al., 2005; Verdoy et al., 2006; Fahmi et al., 2011; Kim and Nam, 2013; Bargaz et al., 2015), very few studies have focused on the effect of exogenous proline. Sabagh et al. (2017) studied salt-stressed *Glycine max* induced by 15 mM NaCl, and supplied 25 mM proline in the growing medium. The result was an increase in nodule number and biomass. Furthermore, the loss in nitrogenase activity caused by salinity was overcome when proline was applied (Sabagh et al., 2017). Similar results were observed in *Cicer arietinum* growing under conditions of cadmium toxicity, where 20 mM exogenous proline alleviated the negative effect of cadmium (25 mg/kg) and increased the number of nodules, the leghemoglobin content and the nitrogenase activity (Alyemeni et al., 2016). Moreover, the positive effect of exogenous proline on nitrogenase activity under salt stress has been reported not only in plants but also in some bacterial strains like *Klebsiella pneumonia* (Le Rudulier et al., 1982). Investigating the relationship between proline metabolism and BNF, Ren et al. (2018) demonstrated that overexpression of *StP5CS* enhanced the relative expression of two nodulation-related genes and one leghemoglobin gene. This was reflected by an increase in nodulation and nitrogen fixation under salt stress. Furthermore, overexpression of *P5CS* from *Vigna aconitifolia* in *M. truncatula* enhanced tolerance to salt stress and improved nitrogenase activity (Verdoy et al., 2006). In addition, Kim and Nam (2013) demonstrated that *P5CS3* regulated *M. truncatula* nodule number under salt stress. The above studies show that exogenous proline may improve BNF under salt stress, but the detailed mechanisms behind this relationship are still not clear as well as its relevance to field conditions.

### Proline Toxicity in Salt-Stressed Plants

Despite the protective roles of exogenous proline on salt-stressed plants, several papers reported that its positive effect is concentration-dependent, high concentration could cause a toxic effect in plants (Hellmann et al., 2000; Maggio et al., 2002). For example, while low concentrations (20–33 mM) alleviated the deleterious effect of salt stress, external supplementation of high proline concentration (50 and 100 mM) was found to be toxic for both salt-stressed and unstressed callus culture of mung bean (Kumar and Sharma, 1989). In agreement with that, Rodriguez and Heyser (1988) demonstrated that 10 mM of exogenous proline seriously inhibited the normal growth of Distichlis suspension cultures under 260 mM of salt stress. Similarly, in salt stressed *Oryza sativa*, while low concentrations (20–30 mM) of proline were effective in mitigating the adverse effect of 100 mM NaCl on growth, higher concentrations (40 to 50 mM) resulted in growth reduction (Roy et al., 1993). In addition, in contrast to 1 mM, the external supplementation of 10 mM of proline to salt stressed *Solanum lycopersicum* decreased leaf and root fresh weights, even leading to plant death if proline is added in high concentration (Heuer, 2003). Furthermore, Rajendrakumar et al. (1997) showed that proline at high concentration could destabilize the DNA helix, lower the DNA melting point, increase susceptibility to S1 nuclease and insensitivity to DNAase1. Interestingly *p5cdh* and *prodh* mutants were shown to be more sensitive to proline treatments (Mani et al., 2002; Nanjo et al., 2003; Deuschle et al., 2004; Cabassa-Hourton et al., 2016), indicating the importance of proline catabolism in the regulation proline level for plants. However, the underlying mechanism of proline toxicity remains elusive.

## Conclusions and Prospects

**Figure 6:**
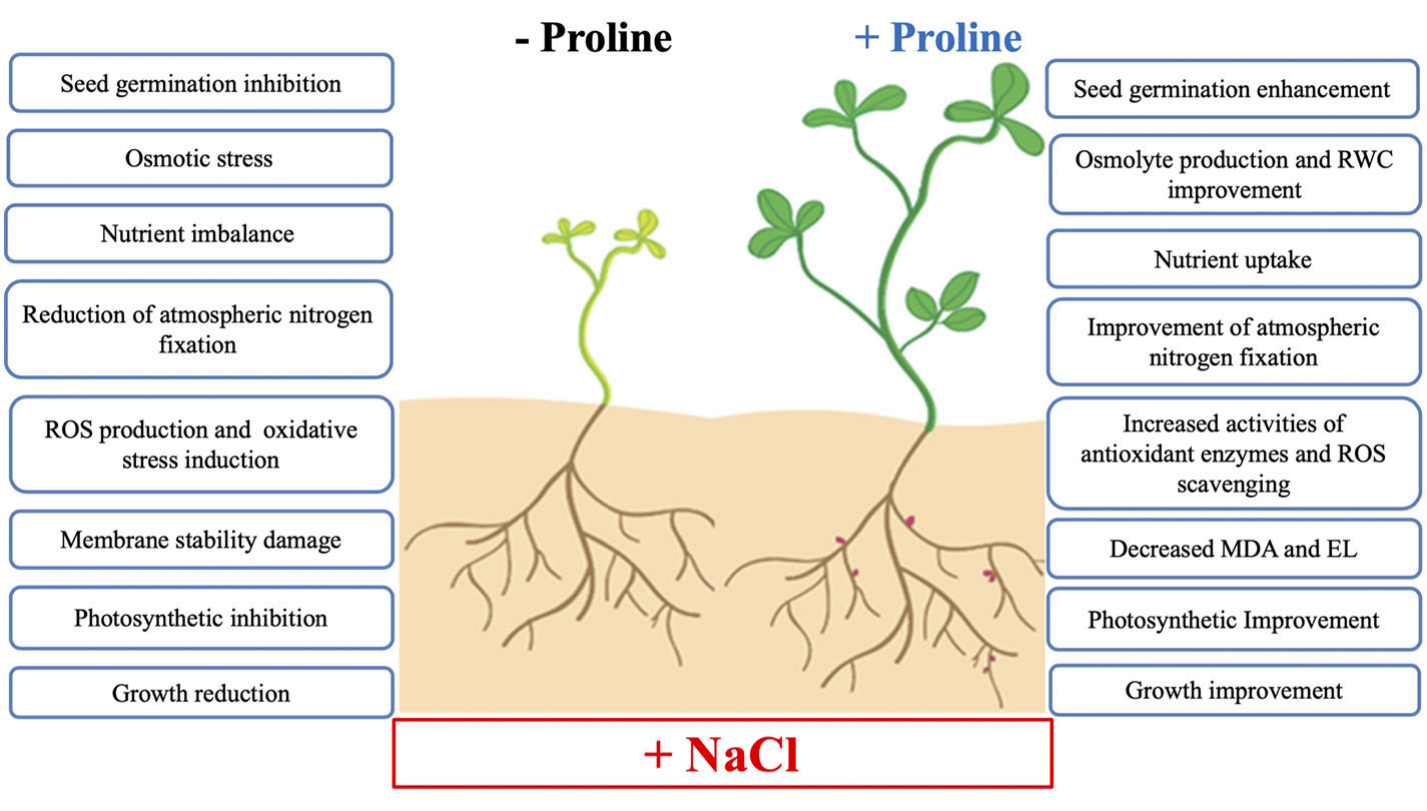
Summary of the main effects of exogenous proline in plant salt tolerance. Abbreviations: RWC, relative water content; ROS, reactive oxygen species; MDA, malondialdehyde; EL, electrolyte leakage.

Exogenous proline application can improve salt tolerance by regulating physiological, biochemical and enzymatic processes and have a positive effect on plant growth, development and productivity under salt stress conditions. To focus on where potential solutions will be found in future crop research, the proposed beneficial effects of exogenous proline on salt stress tolerance in developing plants are summarized in **Figure 6**.

Exogenous proline reduces Na^+^ and Cl^−^ content and increases K^+^/Na^+^ ratio in many plant species (**Table 1**) (*e.g.* Abdelhamid et al., 2013; de Freitas et al., 2018; de Freitas et al., 2019). Na^+^/H^+^ is an antiporter plasma membrane transporter, encoded by an *SOS1* gene, that pumps Na^+^ from root cells to leaves, boosting salt stress tolerance (Zhu and Gong, 2014; Bargaz et al., 2015). High-affinity K^+^ transporter (HKT) is another transporter that mediates salt tolerance in various plant species through regulation of the transport of salt ions from root to shoot (Kaundal et al., 2019; Thouin et al., 2019). In view of the important roles of these two transporters in plant salt tolerance, it would be interesting to investigate how exogenous proline can regulate the *SOS1* and *HKT* gene expression under salt stress and their relationship with salt tolerance.

Water restriction is one of the main effects of salt stress in plants (Farissi et al., 2014). Exogenous proline was widely reported to increase plant water content under salt stress (**Table 1**), and this may contribute to salt dilution and as a result plant growth improvement (Huang et al., 2009; Zheng et al., 2015). Aquaporins are a group of transporters that facilitate absorption of water by plant from soil. Under salt stress, there is a positive correlation between the expression of aquaporin genes and salt tolerance of *Eutrema salsugineum* (Qin et al., 2019). To better understand the mechanism by which exogenous proline improves plant water relations under salt stress, the effect of this osmoprotectant on the expression of aquaporin genes under salt stress will be interesting to investigate.

BNF is an important process that improves soil fertility but it is very sensitive to salt stress from the establishment of the symbiosis to nitrogen fixation (Zahran, 1999; Monica et al., 2013). The ability of exogenous proline to improve nitrogen acquisition under salt conditions was reported in several species (Kaya et al., 2007; Abdelhamid et al., 2013; Alam et al., 2016). The beneficial effect of this molecule in nitrogen nutrition of legumes through nitrogenase activity, however, is poorly understood and very few studies have been done. It will be important to focus on the effect of exogenous proline on nitrogenase gene expression under salt stress to better understand the effect of this multifunctional amino acid on BNF.

The effect of exogenous proline in alleviating the negative impact of salt stress appears to be both dose- and species-dependent. It is still not clear how proline works in reducing the detrimental effect of salt stress and further research is needed. Omics approaches can provide a more holistic molecular perspective of biological systems compared to traditional approaches. Transcriptome analysis has been widely applied to explore genes that are differentially expressed in response to abiotic stresses. These data are essential to identify and potentially manipulate genes that impact stress tolerance under diverse environmental conditions. Increasing amounts of data suggest that proline has certain regulatory functions. Using transcript profiling, Oono et al. (2003) showed that proline can also trigger expression of one third of rehydration-inducible plant genes. Most of the known proline-responsive genes have the conserved PRE cis-acting element in their promoter regions, which is a target of specific bZIP-type transcriptional activators (Oono et al., 2003; Satoh et al., 2004; Weltmeier et al., 2006). From this starting point, the proline-related signaling pathway requires further elucidation using multiomics technologies that dissect the multiple corresponding genes or metabolites. Therefore, further large-scale analyses of transcript, protein and metabolite responses are required to understand how plants respond to proline and the adaptive value of proline in plant stress adaptation.

## Author Contributions

AEM proposed and wrote the review. CC-H and MF commented on the content of the review and revised the text. AS revised the text at different stages of the writing process and contributed to the final version of the manuscript. All authors contributed to the article and approved the submitted version.

## Funding

This work was supported by the Hubert Curien Maghreb Partnership—PHC Maghreb, No. 19MAG41/41482RL—governed by the agreement signed between the French Ministry of Europe and Foreign Affairs and the Algerian, Moroccan and Tunisian Ministries of Higher Education and Scientific Research.

## Conflict of Interest

The authors declare that the research was conducted in the absence of any commercial or financial relationships that could be construed as a potential conflict of interest.
